# Edge area metric complexity scoring of volumetric modulated arc therapy plans

**DOI:** 10.1016/j.phro.2021.02.002

**Published:** 2021-03-06

**Authors:** Julia Götstedt, Anna Bäck

**Affiliations:** a Department of Radiation Physics, Institute of Clinical Sciences, Sahlgrenska Academy, University of Gothenburg, 413 45 Gothenburg, Sweden; b Department of Therapeutic Radiation Physics, Medical Physics and Biomedical Engineering, Sahlgrenska University Hospital, 413 45 Gothenburg, Sweden

**Keywords:** Complexity, Complexity metric, EAM, Quality, Quality control, VMAT

## Abstract

**Background and purpose:**

Aperture-based complexity metrics have been suggested as a method to score complexity of volumetric modulated arc therapy (VMAT) plans. The purpose of this study was to evaluate the edge area metric (EAM) for clinical VMAT plans on a control point and treatment plan level.

**Materials and methods:**

EAM on a control point level was evaluated based on film measurements of 18 static beam openings originating from VMAT plans. EAM on a treatment plan level (arithmetic mean value of EAM scores for control points) was evaluated based on measurements with the Delta^4^® for 200 VMAT plans for four different treatment sites: pelvic, thorax, head and neck, and prostate. Measurements were compared to calculations and dose difference and gamma pass rates were evaluated.

**Results:**

EAM scores on a control point level correlated with Pearson’s r-values of −0.96 and −0.77 to dose difference and gamma pass rates, respectively. The prostate plans had the highest average EAM score. A connection between smaller PTVs and higher EAM scores was found. No correlation between the evaluation result and EAM on a plan level was found.

**Conclusions:**

EAM on a control point level was shown to correlate to the difference between measured and calculated 2D dose distributions of clinical VMAT beam openings. No correlation was found for EAM on a plan level for clinical treatment plans.

## Highlights

• Edge area metric correlated to dosimetric evaluations of control point beam openings.
• Distribution of edge area metric complexity scores were dependent on treatment site.
• No correlation for mean edge area metric and evaluations of full treatment plans.

## 1 Introduction

Volumetric modulated arc therapy (VMAT) involves simultaneous alteration of the beam opening formed by multi-leaf collimators (MLC), gantry speed, and dose rate, for beam modulation during treatment [1]. A treatment field, i.e. arc, is divided in control points for which the MLC leaf positions, gantry angle, and cumulative number of monitor units (MU) are defined.

Treatment plans with high amount of complex beam openings, i.e. small and/or irregularly shaped, will create relatively large regions that lack charged particle equilibrium where clinical dose calculation algorithms have difficulties to accurately calculate the dose distribution [2]. Furthermore, complex beam openings make the delivered dose more sensitive to small variations in the treatment machine settings, e.g. the positioning of the MLC leaves [3], [4]. Therefore, treatment plans with high amount of complex beam openings might lead to clinically relevant dose differences between planned and delivered dose distributions [2], [3], [4], [5].

Aperture-based complexity metrics have been suggested to score the complexity of VMAT plans to detect complexity at an early stage of treatment preparation for either re-consideration, re-planning, or extra attention during quality controls (QC) [6], [7]. A complexity metric could also be integrated in the objective function of the optimization process in the TPS to automatically make treatment plans less complex [8], [9].

Before making clinical decisions based on a complexity metric, thorough evaluation of the metric should be performed. Numerous comparative studies of different complexity metrics and their correlation to measurement results of commonly used pre-treatment QC methods have been performed [6], [7], [10], [11], [12], [13], [14], [15]. However, dedicated evaluations of metrics with the purpose to increase information on the dosimetric uncertainty of a plan beyond common QC results, are scarce.

The edge area metric (EAM) has shown promising results in a previous evaluation study where different complexity metrics were compared based on artificially generated static beam openings [16]. To our knowledge, EAM scores have not yet been evaluated for clinical VMAT beam openings. The purpose of this study was to evaluate EAM for clinical VMAT plans on a control point and treatment plan level for different treatment sites.

## 2 Material and method

EAM is based on dividing a beam opening into a complex (*R_1_*) and a non-complex (*R_2_*) region and is defined as EAM = R_1_/(R_1_ + R_2_) [16] (Supplementary Fig. 1). The complex region encloses an area both inside and outside of the beam opening edge and the non-complex region is defined as the rest of the open area within the beam opening. Hence, EAM score is a positive value up to 1 for the most complex openings. Besides the absolute level on the EAM scale, only small relative variations in EAM have been observed when varying the extent of R_1_ from 1 mm to 5 mm (at isocenter distance). The largest separation between lowest and highest EAM scores for control points originating from the same treatment was found for R_1_ of 2.5 mm which was used in this study. The EAM calculations were performed using an in-house developed MATLAB® software.

VMAT plans used in this study were approved and used for treatments within four groups of treatment sites: larger targets in the pelvic, targets in the thorax and the head and neck (H&N) region, and prostate cancer treatments. This project was classified as a quality development and assurance project without processing of personal data and no statement of access to use the treatment plans was required. The plans were created for Clinac iX or TrueBeam STx (Varian Medical Systems) equipped with Millennium and High Definition MLC respectively. Dose calculations were performed using analytical anisotropic algorithm (AAA) in Eclipse (version 13.6) with calculation grid size 0.25 cm × 0.25 cm. The minimum leaf gap was set to 0.05 cm and a full arc (358 deg) were divided in 178 control points. In general, the treatment plans for prostate cancer and larger targets in the pelvic region included 2–3 full arcs while the plans created for targets in the H&N and thorax region were more diverse but, in most cases included 2–3 either full or partial arcs.

### 2.1 EAM for VMAT control points

Six full arc treatment plans – two H&N plans (two arcs each), three prostate plans (one single and two double arc plans) and one plan created for treatment of vulvar cancer (3 arcs) – were selected for evaluation of EAM on a control point level. From each plan, beam openings from three control points in one of the arcs were selected (Table 1). The beam openings were selected to cover the whole range of EAM scores for the plans (0.35 to 0.99). Beam openings with the same EAM score originating from different plans were also selected. Examples of beam openings are given in Supplementary material (Supplementary Fig. 2).

**Table 1:** Information about the 18 beam openings for evaluation of EAM on a control point level. The beam openings are grouped according to their EAM score. The MLC type is either Millennium (M) or High Definition (HD).

Beam opening number	EAM score	Treatment machine	MLC type	Treatment site	Beam opening area [cm^3^]	Number of sub openings
1	0.35	Clinac iX	M-MLC	Pelvic	109.3	2
2	0.35	Clinac iX	M-MLC	H&N	120.8	3

3	0.41	TrueBeam STx	HD-MLC	H&N	85.5	3
4	0.41	TrueBeam STx	HD-MLC	Prostate	26.8	1
5	0.41	TrueBeam STx	HD-MLC	Prostate	29.9	1

6	0.55	Clinac iX	M-MLC	Pelvic	55.0	8
7	0.55	Clinac iX	M-MLC	H&N	63.0	3
8	0.55	Clinac iX	M-MLC	Prostate	24.9	2

9	0.74	TrueBeam STx	HD-MLC	H&N	44.6	8
10	0.74	Clinac iX	M-MLC	Pelvic	57.5	7
11	0.74	Clinac iX	M-MLC	H&N	39.8	6
12	0.74	TrueBeam STx	HD-MLC	Prostate	16.6	2

13	0.87	TrueBeam STx	HD-MLC	H&N	25.7	10
14	0.87	Clinac iX	M-MLC	Prostate	14.5	6
15	0.87	TrueBeam STx	HD-MLC	Prostate	14.2	2

16	0.99	Clinac iX	M-MLC	Prostate	6.4	10
17	0.99	TrueBeam STx	HD-MLC	Prostate	9.0	7
18	0.99	TrueBeam STx	HD-MLC	Prostate	3.7	12

The dose distribution of the beam openings was measured at the respective treatment machine using Gafchromic EBT3 films. The double exposure measurement procedure used, with pre-scan and pre-exposure, is described previously [16]. The measurements were performed with the gantry in 0 degrees and the film at 10 cm depth in a solid water phantom with a source-surface-distance (SSD) of 90 cm and calculated in the same geometry. The number of MU was adjusted to result in a calculated maximum dose of 2 Gy (±0.2%) at 10 cm depth. Each measurement was repeated on three different occasions and the result is reported as the mean value of these measurements to account for variations of sensitivity within and between separate film sheets and other uncertainties related to the measurement and evaluation procedure as well as to account for treatment machine delivery variations.

The measured and calculated dose distributions were imported into the evaluation software RIT113 (Radiological Imaging Technology©). The hair cross from the measurement setup was marked in the margin of the film and used for rotation correction and to define the isocenter in the measured dose distributions. The isocenter was used for registration to the corresponding calculated dose distributions. This alignment method is independent of potential inaccuracies in the dose calculation due to a non-proper modelling of the tongue and groove design of the MLC leaves which would shift the position of the penumbra in a direction opposed to the MLC leaf direction. The median dose value within a centrally placed region of interest (0.5 cm × 0.5 cm or 0.25 cm × 0.25 cm for the three smallest openings) in both measured and calculated dose distributions were used for normalization. The calculated and measured dose distributions were compared pixel-by-pixel with a global dose difference criterion of 5% and with gamma evaluation using 3%, 1 mm criterion. A stricter criterion than 5%, without including a DTA criteria would be in the range of the inherent uncertainty of the measurement procedure. The EAM scores were compared to the relative number of pixels within the evaluation criteria (threshold of 10% of the maximum calculated dose).

### 2.2 EAM for VMAT plans

The arithmetic mean value of the EAM scores calculated on a control point level was evaluated for scoring complexity on a treatment plan level. Control points included in avoidance sectors with zero dose rate were excluded.

EAM scores were calculated for 200 of the most recent VMAT plans (50 plans for each of the four treatment sites) and analyzed on both a treatment plan and on a control point level. The design of EAM implicitly means that a beam opening with a smaller open area has a higher EAM and the beam opening area is often related to the projection size of the planning target volume (PTV). To study this relation, the PTV volumes were extracted. In the case of multiple PTVs, the union of all PTV volumes was extracted.

The plans were measured on one occasion before treatment start using the Delta^4^® (ScandiDos) phantom described previously [17], [18], [19]. A half arc with a 10 cm × 10 cm beam opening formed using the collimator jaws was also measured to correct for the daily machine output variation. The dose distributions were aligned using the optimized phantom position tool provided in the Delta^4^® software. The calculated and measured dose were compared in the diode measurement points. The pass rate for the evaluated points (receiving higher than 20% of the maximum dose) within a global dose difference of 3% normalized to the maximum dose in the measurements, as well as within a gamma criterion of 3%, 1 mm, were compared to EAM on a plan level. A stricter criterion would be in the range of the dosimetric accuracy [18].

## 3 Results

### 3.1 EAM for VMAT control points

**Fig. 1:**
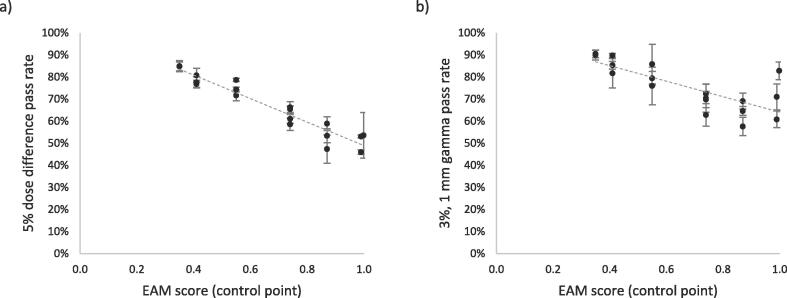
The calculated EAM complexity scores for the beam openings of the 18 selected VMAT control points versus a) the 5% dose difference pass rate and b) 3%, 1 mm gamma pass rate. The evaluation result for each point is the mean value of three film measurements and the error bars plotted in the figure are the standard deviations from the three measurements. A linear trend line is fitted to the result.

The linear correlations, expressed as Pearson’s r-values, between EAM scores and evaluated differences between measurements and calculations on a control point level were −0.96 and −0.77 for dose difference and gamma pass rates respectively (Fig. 1). The spread in mean dose difference and gamma pass rates for beam openings from different treatment plans but with the same EAM score was larger for more complex beam openings with higher EAM (Fig. 1 and Supplementary Table 1).

### 3.2 EAM for VMAT plans

The average EAM score for all plans was 0.59 and 0.67, 0.59, 0,58 and 0,52 for prostate plans, thorax plans, H&N plans, and plans planned for larger targets in the pelvic, respectively. The proportion of the prostate plans with an EAM score higher than the total average (0.59) was 94% and the corresponding proportion for the plans planned for larger targets in the pelvic was 6%.

**Fig. 2:**
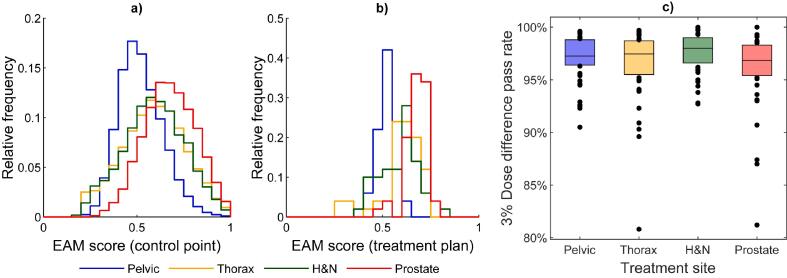
The complexity analysis of the 200 VMAT plans is illustrated for four treatment sites (50 plans in each group) as a) the relative frequency of EAM scores on control point level, b) the relative frequency of EAM scores on treatment plan level and c) the 3% dose difference pass rate results for the evaluated Delta^4^ measurements and AAA calculations. The area under the curves in the frequency histograms are normalized to 1. The boxes in c) are defined as the data within the 25th and 75th percentiles, and the median values of respective dataset is marked with a horizontal line.

**Fig. 3:**
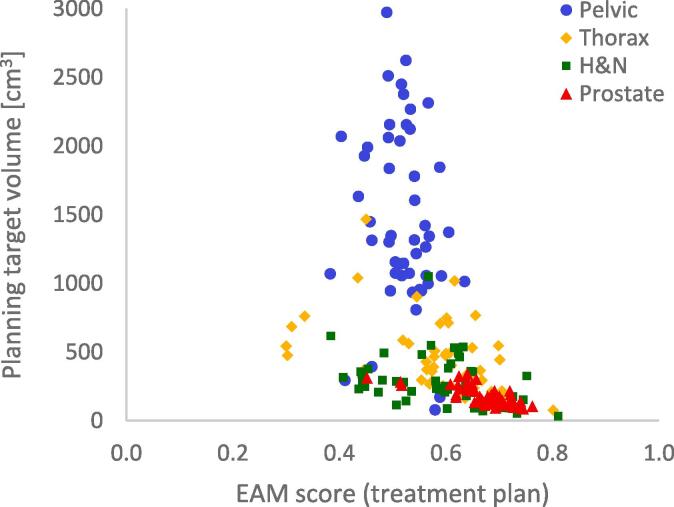
The planning target volume (PTV) (the union volume when multiple PTVs are defined) for 200 patients and the EAM scores calculated on a treatment plan level for the corresponding VMAT plans studied. The results are separated for the group of patients treated for larger targets in the pelvic region (blue circles), thorax treatments (yellow diamonds), H&N treatments (green squares) and prostate treatments (red triangles). (For interpretation of the references to colour in this figure legend, the reader is referred to the web version of this article.)

The EAM results per treatment site on a plan level were confirmed by the results of EAM on a control point level (Fig. 2). The higher complexity found for prostate plans was also indicated from the dose difference pass rate analysis shown for comparison (Fig. 2c). On average, the prostate plans had a lower EAM for the lateral beam openings as seen in polar plots for EAM as a function of gantry angle (Supplementary Fig. 3). No such average trends could be seen for the other treatment sites. The group of prostate cancer patients included a larger number of patients with smaller PTVs compared to the other three groups. A connection between smaller PTVs and higher EAM scores was found (Fig. 3).

**Fig. 4:**
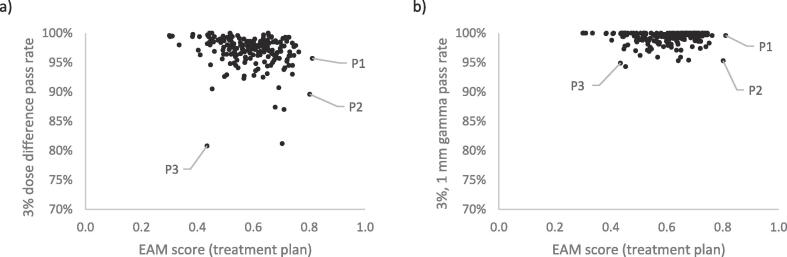
Scatter plots for the EAM scores on a treatment plan level (mean value of EAM scores on a control point level) and a) the 3% dose difference pass rate and b) 3%, 1 mm gamma pass rate for the evaluated Delta^4^ measurements and AAA calculations. Three plans are identified to be subject for further investigation. Two plans (P1, P2) due to the high EAM scores and one plan (P3) due to the combination of a low dose difference pass rate and low EAM score.

**Fig. 5:**
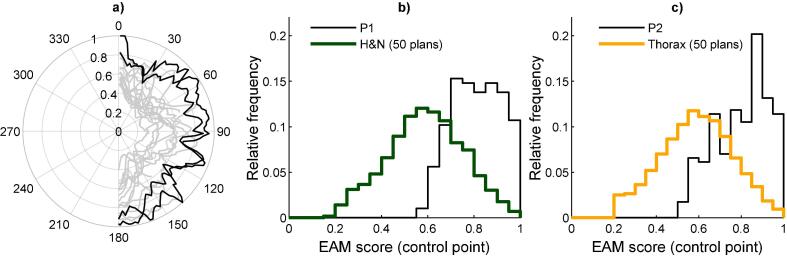
a) The two arcs of the P1 H&N plan (from Fig. 4) is plotted as a polar plot with the EAM scores on a control point level (r-axis) plotted for corresponding gantry angle (-axis) as black lines compared to 6 H&N plans using the same treatment arcs plotted as grey lines. b) The P1 H&N plan is shown in a frequency histogram for EAM scores on a control point level for comparison with the EAM scores on a control point level for all 50 H&N plans. c) The P2 thorax plan for EAM on a control point level is compared to all 50 thorax plans. The area under the curves in the frequency histograms are normalized to 1.

No linear correlation was found between EAM scores and evaluated differences between measurements and calculations on a treatment plan level (Fig. 4). Two plans, P1 and P2 (Fig. 4), stood out with respect to high EAM scores of 0.81 and 0.80 respectively. P1 was planned for treatment of a small PTV (32 cm^3^) in the H&N and P2 was planned for a postoperative treatment in the thorax region (PTV 76 cm^3^). Comparison of EAM on a control point level for P1 and P2 to the other plans of respective treatment site showed that the P1 and P2 histograms were shifted towards higher complexity (Fig. 5). The increased complexity of the P1 plan was found for almost all separate gantry angles when compared to 6 H&N plans planned for the same angles (Fig. 5a).

## 4 Discussion

The performance of EAM was evaluated on a control point and on a treatment plan level for VMAT plans. A correlation of EAM scores and dose differences between measured and calculated dose distributions was found on control point level. The possibility to evaluate complexity on control point level to provide information of complexity variations as a function of gantry angle can be advantageous in the treatment plan evaluation process. No correlation to differences between calculated and measured dose was found when the mean value of EAM (on control point level) was used to quantify the complexity on treatment plan level. The EAM scores were found to be treatment site dependent and the prostate plans were found to have the highest average EAM.

The measurement depth of 10 cm represents a relevant tumor depth and has been used in other studies [8]. The evaluated pass rates are expected to be different at other depths but the relation to EAM is expected to be similar. However, because dose differences are expected to be more pronounced at shallower depths, evaluations at one single depth is a limiting factor. The calculation grid size has an influence on both the pass rates and the correlation to EAM [20] and the grid size was chosen based on common clinical use. For static fields with the same EAM score, the spread in pass rates were larger for the group of fields that were ranked as more complex. This can be explained by an increased uncertainty that affects the variations in the delivered dose for different occasions but also the sensitivity to measurement uncertainties. This supports the argument that it is important to decrease the amount of complex beam openings to create high quality and robust treatment plans.

An evaluation method including a DTA criteria, such as the gamma evaluation, can be used to disregard some dose differences due to systematic and random measurement errors. However, a DTA criterion will also disregard true differences between calculated and delivered dose. In fact, commonly used evaluation methods which includes a DTA criterion has been shown to be an insufficient tool for the purpose of detecting differences between calculated and measured dose distributions [6], [7], [11], [21], [22], [23], [24], [25]. The evaluation of dose differences in the penumbra region is challenging since a relatively large dose deviation in a specific point could be due to a relatively small alignment error. Evaluations that exclude a DTA criterion therefore need careful alignment methods but will nevertheless lead to larger deviations, especially in high dose regions, which is a limitation. The choice of evaluation criteria for gamma evaluation is critical [25], [26], [27], [28] and should be selected based on the specific purpose. The criteria must also be selected pursuant to the inherent characteristics of the calculation and measurement procedures [25], [29]. Evaluations based on dose difference alone are expected to result in lower pass rates as compared to gamma evaluations but will also result in a higher differentiation of treatment plans (Fig. 4).

The prostate plans had the highest average EAM score, probably mainly due to the smaller PTV. In our experience, a beam opening originating from a prostate plan can be quite irregular despite the regular form of the target (Supplementary Fig. 2). The complexity is dependent on the number of arcs used in the treatment plans [12] and on the TPS and treatment machine [30]. Thus, a complexity score should be interpreted in relation to a baseline of plans for the same treatment site, technique, TPS, and treatment machine.

One plan (P3) stood out with a low pass rate but at the same time a low EAM (Fig. 4a). This plan was created to treat Hodgkin’s lymphoma with three anterior partial VMAT arcs. The calculated dose in the Delta^4^ geometry resulted in several high dose gradient regions which might have led to an increased measurement uncertainty. The PTV (1040 cm^3^) was relatively large in comparison to the other patients (mean 590 cm^3^, range 30 to 2970 cm^3^) which partly explains the low EAM.

EAM has been categorized as an accuracy metric [7]. Other examples of accuracy metrics evaluated for VMAT are based on the opening area alone, “segment area per control point” [12], and “small aperture score” [11], based on the irregularity of the opening alone, “leaf offset impact on calculation” [15], while some, like EAM, combines area and irregularity of the opening, “converted aperture metric” [16], and “edge metric” [8], [13]. The dynamic nature of the VMAT delivery can also lead to an increased uncertainty which is not taken into account by the purely aperture-based accuracy metrics. The “comprehensive modulation index” [31] was categorized as a combination of an accuracy and a deliverability metric.

Even though a correlation between EAM and dosimetric evaluations was found for VMAT control point beam openings it does not directly translate to a correlation on treatment plan level. One reason could be that the mean value blur information detected by EAM on a control point level. It might also be related to limitations in the quasi-3D measurement method used. Lack of correlation on a plan level for accuracy metrics has also been reported by others [10], [11], [12], unless evaluating individual structure volumes separately [12]. Our study included exclusively plans that were approved for treatment. Other studies that included both plans that passed and failed the clinical QC were able to show that accuracy metrics could distinguish plans that failed from those that passed [13], [15]. All this underlines the importance of defining the purpose of a complexity metric and to validate that the metric meets that specific purpose.

To interpret the clinical relevance of aperture-based complexity, the dose contribution should be considered. Some suggested VMAT complexity metrics include a MU weighting factor [13], [14], [24], [30]. Introducing a weight parameter to decrease the impact of control points with lower MUs raises the question of the most suitable relation between that weight and the number of MUs. For example, a highly complex beam opening needs a higher number of MUs to deliver the same dose compared to a less complex beam opening, but a highly complex beam opening often has a smaller area and consequently contributes to a smaller volume. Furthermore, the clinical impact depends on the combination of the amount of dose and location within the patient. The translation from MU to clinically relevant dose contribution is not straightforward. Interpretation of patient-specific clinical relevance of plan complexity is studied in a parallel work by our group [32].

In conclusion, the EAM has been shown to correlate to differences between measured and calculated dose distributions for clinical VMAT control points and can be used to quantify the variation of complexity in different parts of the treatment arc. The EAM complexity score should be interpreted in relation to the treatment site. The complexity on treatment plan level quantified as a mean value of EAM control point scores was found to not correlate to differences between measured and calculated dose distributions of clinical VMAT plans.

## Declaration of Competing Interest

The authors declare that they have no known competing financial interests or personal relationships that could have appeared to influence the work reported in this paper.
