# Supplementary material for: Edge area metric complexity scoring of volumetric modulated arc therapy plans
Source: Phys Imaging Radiat Oncol. 2021 Mar 6;17:124–9. doi: 10.1016/j.phro.2021.02.002 (PMC8058026; doi:10.1016/j.phro.2021.02.002)
Supplement: Supplementary data 1 [file mmc1.pdf]

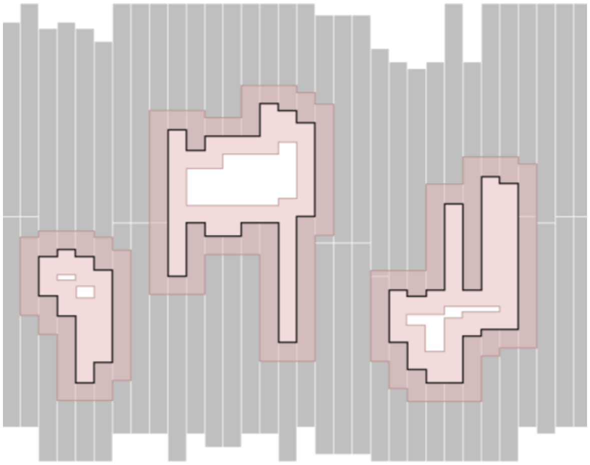

Supplementary figure 1. Illustration of a control point with a beam opening separated in three sub openings. The beam opening MLC edge is marked as a black line and the complex region R_1 is illustrated as the transparent red area enclosing a region on both sides of the MLC edge. The non-complex region R_2 is defined as rest of the open area within the beam opening (white region).
