# Supplementary material for: Edge area metric complexity scoring of volumetric modulated arc therapy plans
Source: Phys Imaging Radiat Oncol. 2021 Mar 6;17:124–9. doi: 10.1016/j.phro.2021.02.002 (PMC8058026; doi:10.1016/j.phro.2021.02.002)
Supplement: Supplementary data 2 [file mmc2.pdf]

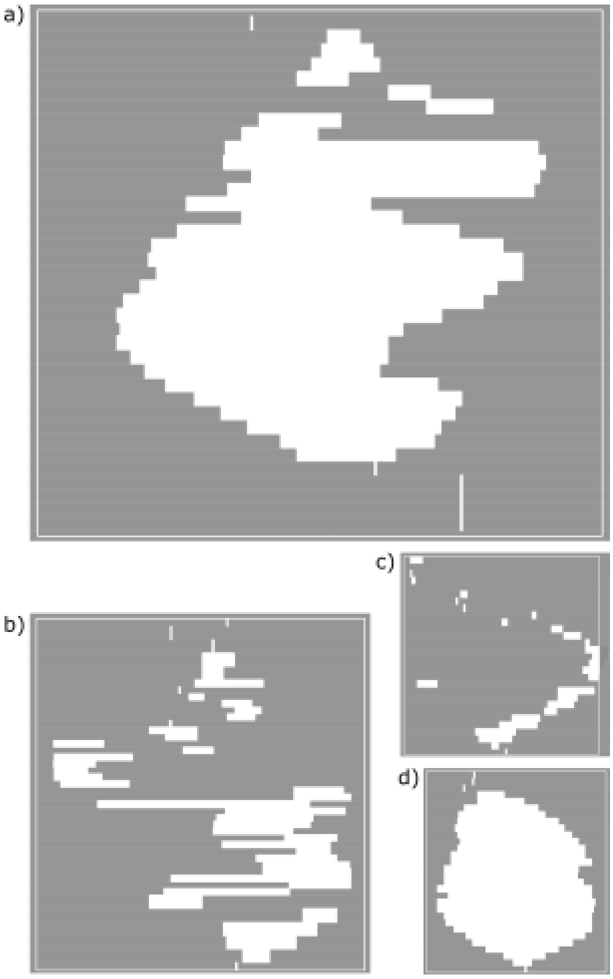

Supplementary figure 2. Examples of beam openings including one of the highest and lowest EAM scores for the control points originating from the H&N and prostate plans that were selected for the evaluation of EAM on a control point level. a) H&N beam opening with an EAM of 0.35, (Beam opening 2 in table 1). b) H&N beam opening with an EAM of 0.87 (Beam opening 13 in table 1). c) prostate beam opening with an EAM of 0.99 (Beam opening 18 in table 1) and d) prostate beam opening with an EAM of 0.41 (Beam opening 4 in table 1). The beam openings are comparable in size as they are the same scale in this figure.
