# Supplementary material for: Edge area metric complexity scoring of volumetric modulated arc therapy plans
Source: Phys Imaging Radiat Oncol. 2021 Mar 6;17:124–9. doi: 10.1016/j.phro.2021.02.002 (PMC8058026; doi:10.1016/j.phro.2021.02.002)
Supplement: Supplementary data 3 [file mmc3.pdf]

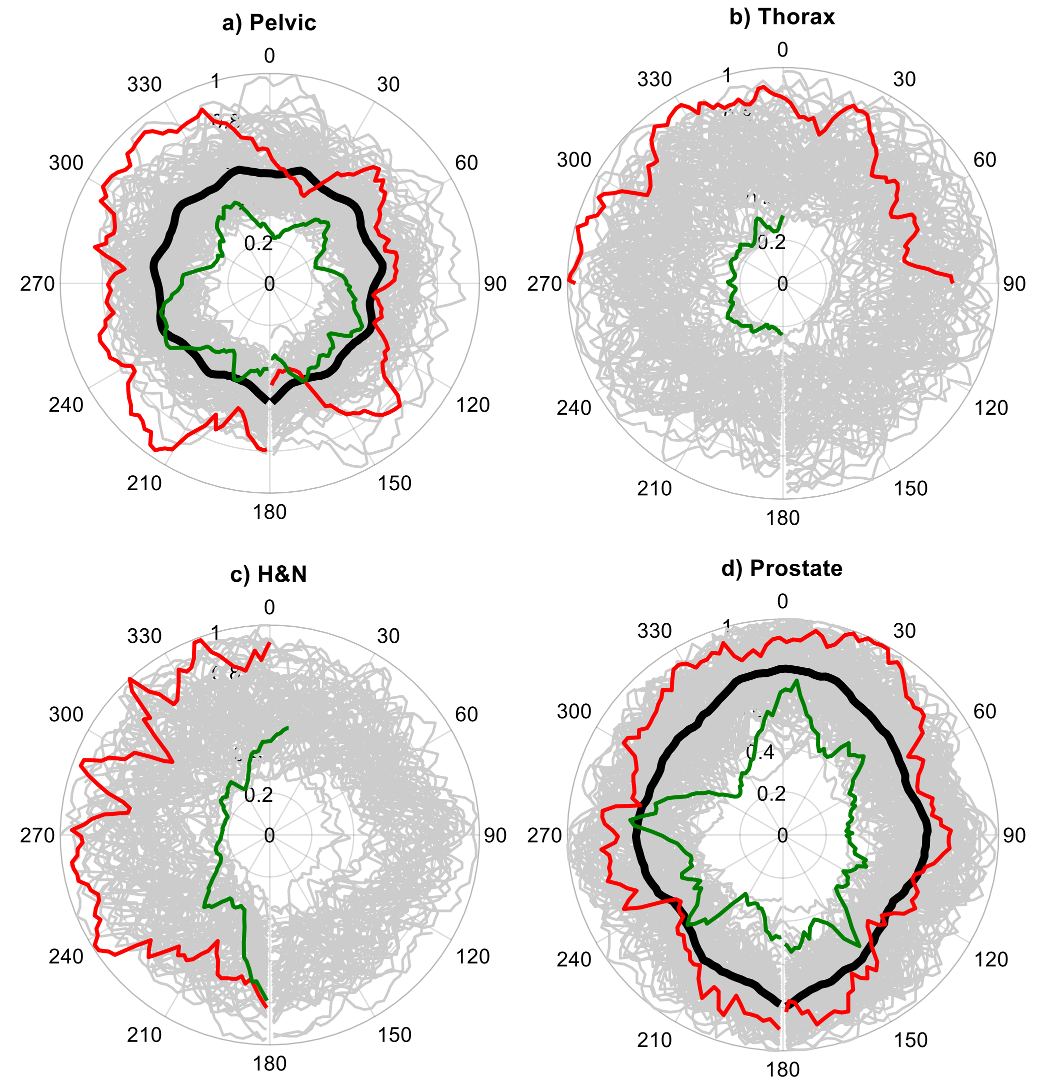

Supplementary figure 3. Complexity level calculated as EAM score as a function of gantry angle for the four diagnose groups studied a) pelvic, b) thorax, c) H&N and d) prostate. All arcs are outlined in the polar plots as thin grey lines. The arc with the highest (red line) and lowest (green line) mean EAM score for an individual arc is outlined for each diagnose group. The treatment plans planned for the pelvic region (a) and (d) are planned with full arcs and the mean EAM score per gantry angle is defined in the polar plots with a thick black line. The treatments for thorax (b) and H&N (c) are planned using different gantry angles depending on the location of the target and a mean EAM score for a specific gantry angle will not be meaningful.
