# Supplementary material for: Edge area metric complexity scoring of volumetric modulated arc therapy plans
Source: Phys Imaging Radiat Oncol. 2021 Mar 6;17:124–9. doi: 10.1016/j.phro.2021.02.002 (PMC8058026; doi:10.1016/j.phro.2021.02.002)
Supplement: Supplementary data 4 [file mmc4.docx]

Supplementary Table 1. The spread in mean dose difference and gamma pass rates for beam openings from different treatment plans but with the same EAM score is listed in the table. The evaluation criteria used were 5% dose difference and 3%, 1mm gamma evaluation. The spread in pass rates for the five complexity levels is expressed as one standard deviation. The complexity level with an EAM score of 0.74 included four different static beam openings and the other levels included three different static beam openings. No standard deviation was calculated for the EAM scores of 0.35 because this group included only two beam openings and where therefore not included in the table.

| Complexity level  EAM score | Dose difference evaluation | Gamma evaluation |
| --- | --- | --- |
| 0.41 | 1.7 | 3.3 |
| 0.55 | 2.9 | 4.1 |
| 0.74 | 3.2 | 3.6 |
| 0.87 | 4.7 | 4.7 |
| 0.99 | 3.5 | 9.0 |
